# Compact SPAD-Based Pixel Architectures for Time-Resolved Image Sensors

**DOI:** 10.3390/s16050745

**Published:** 2016-05-23

**Authors:** Matteo Perenzoni, Lucio Pancheri, David Stoppa

**Affiliations:** 1 Integrated Radiation and Image Sensors, Fondazione Bruno Kessler, Trento 38123, Italy; stoppa@fbk.eu; 2 Department of Industrial Engineering, University of Trento, Trento 38123, Italy; lucio.pancheri@unitn.it

**Keywords:** single-photon avalanche diode, SPAD, time-resolved imaging, time-gating photon counting

## Abstract

This paper reviews the state of the art of single-photon avalanche diode (SPAD) image sensors for time-resolved imaging. The focus of the paper is on pixel architectures featuring small pixel size (<25 μm) and high fill factor (>20%) as a key enabling technology for the successful implementation of high spatial resolution SPAD-based image sensors. A summary of the main CMOS SPAD implementations, their characteristics and integration challenges, is provided from the perspective of targeting large pixel arrays, where one of the key drivers is the spatial uniformity. The main analog techniques aimed at time-gated photon counting and photon timestamping suitable for compact and low-power pixels are critically discussed. The main features of these solutions are the adoption of analog counting techniques and time-to-analog conversion, in NMOS-only pixels. Reliable quantum-limited single-photon counting, self-referenced analog-to-digital conversion, time gating down to 0.75 ns and timestamping with 368 ps jitter are achieved.

## 1. Introduction

Solid-state image sensors with nanosecond and sub-nanosecond timing resolution are needed in applications such as optical ranging, fluorescence microscopy and Raman spectroscopy [1,2,3]. Research is moving in two main directions: on the one hand lock-in pixels with high shutter efficiencies and high frequency operation are already used in 3D Time-of-Flight cameras [4]. On the other hand, time-resolved pixels based on single-photon avalanche diodes (SPADs) appear more and more a feasible and competitive perspective.

The last years have seen CMOS SPAD-based sensors enter the consumer market with products based on single-pixel detectors or small arrays. For imaging applications, however, the requirements are much more demanding and several technological challenges still need to be tackled. In addition to device optimization, the use of SPADs in image sensors imposes additional requirements such as yield, uniformity of breakdown voltage and photon detection efficiency (PDE), reduction of optical cross-talk and minimization of guard rings.

Several challenges also need to be solved with respect to the readout electronics. Storing the timing information at the pixel level requires either fast time-gating or in-pixel time-tagging. In addition, especially for large arrays, the pixels should store timing information on multiple photons, to reduce the bandwidth needed for the array readout. These operations should be performed with a minimum area overhead in order to maintain a small pixel pitch and a good fill factor.

As happened in CMOS image sensor development, the first proof-of-concept designs have exploited standard CMOS processes [5,6,7,8]. Although not optimized in many respects, CMOS proved to be a good platform to test the feasibility of time-resolved image sensors at the architectural level. As the focus passes from proof-of-concept to applications, the optimization of fabrication technologies is unavoidable. Currently, the possibilities for commercial exploitation of CMOS SPAD arrays are increasing and new companies are entering the field, proposing solutions based on customized technologies [9]. Optimized single-photon image sensors will not only be appealing for time-resolved imaging, but also for low-light level imaging, for example in security and scientific applications.

This paper reviews the work on SPAD pixel arrays in the recent years, focusing on pixel architectures suitable for the realization of large pixel arrays. Section 2 presents an overview of the main SPAD characteristics, with particular attention to array integration challenges. In Section 3, several pixel architectures are analyzed and discussed. Finally, a roadmap for future developments is traced.

## 2. SPADs in CMOS Technologies

### 2.1. SPAD Structure

Since their first proof-of-concept in the early 2000s, CMOS SPAD detectors have been integrated in many technology nodes, using both standard, High Voltage or CIS processes. Most of the devices demonstrated so far are based on a p+/nwell junction, which is intrinsically isolated from the p-type substrate [10,11,12]. While the guard ring can be implemented in different ways, the most common embodiments use a deep nwell to separate the guard ring from the substrate. Different guard ring solutions are reviewed in [13].

**Figure 1:**
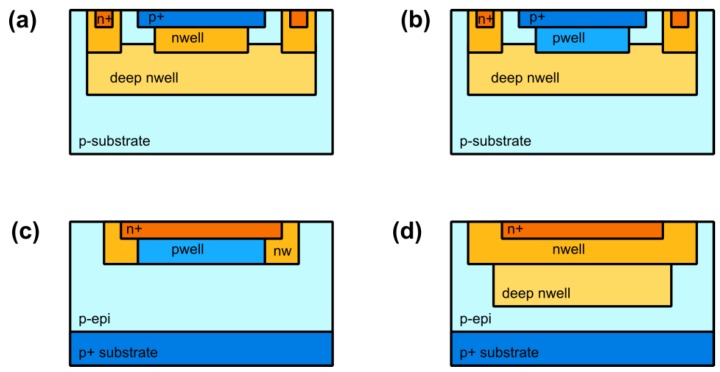
Cross sections of different CMOS SPAD devices (**a**) p+/nwell; (**b**) pwell/deep-nwell; (**c**) n+/pwell; (**d**) deep-nwell/p-epi/p+ sub.

The immediate advantage of this configuration is the possibility of accessing both the anode and cathode, and thus the direct coupling to the readout electronics operating at low voltage. To obtain the structure in Figure 1a, only an additional deep-nwell implantation is needed in addition to the layers typically available in a standard CMOS. A higher red and NIR sensitivity can be obtained with a deeper junction, for example a pwell/deep-nwell, as shown in Figure 1b [12,14].

N-in-p device structures have been proposed, with the active junction based on a or n+/pwell junction or a deep-nwell/p-epi/p+sub (Figure 1c,d) [15,16]. The last options potentially offer an increased photon detection efficiency (PDE) in the red and NIR spectral region, but require a quenching resistor at the high voltage node and a capacitive decoupling of the readout circuit. Their use in a pixel is thus not straightforward.

### 2.2. Figures of Merit

The excellent timing resolution, in the picosecond range, is one of the main advantages of SPAD devices. It has been demonstrated that even unoptimized processes can lead to devices having a jitter of few tens of ps FWHM [17]. One of the key factors to minimize the device jitter is the reduction of photo-carrier diffusion towards the high-field region. To this extent, p+/nwell structures typically feature a very low jitter, as the diffusion tail is often reduced to a minimum, while the jitter is larger in pwell/deep-nwell devices [14,18].

**Figure 2:**
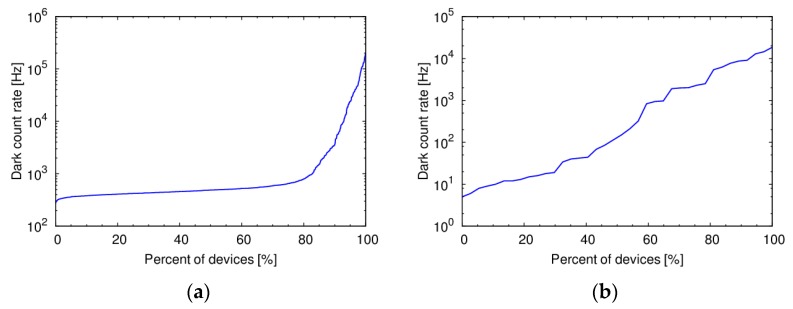
Dark Count Rate distribution of SPADs fabricated in different CMOS process technologies (**a**) 0.35 μm High Voltage with 130-μm^2^ active area [19]; (**b**) 0.7 μm High Voltage with 100-μm^2^ active area [11].

The dark count rate (DCR) is usually the most important source of noise in SPADs, since its fluctuations set the minimum detectable light signal. In a SPAD array, even though all the devices are nominally equal, DCR can have an enormous variability, spanning several orders of magnitude (Figure 2). What is really important in arrays is therefore not the DCR of a single device, but the whole distribution. If small SPADs are considered, two common types of distribution are typically found. A first case where most of the devices have a similar DCR, while a small percentage have large value, as shown in Figure 2a. It is not infrequent to find distributions where a plateau does not exist, as in Figure 2b. The different behaviors can be explained by considering the dominant sources of DCR for different SPAD structures.

**Figure 3:**
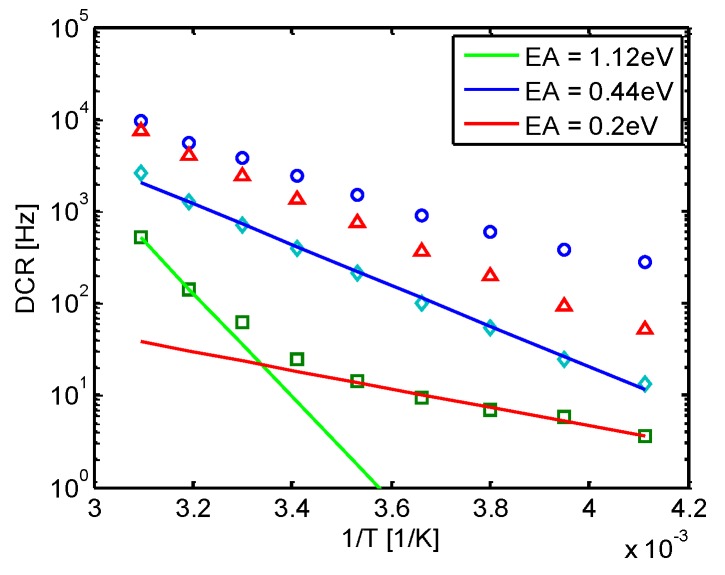
DCR temperature dependence for 10-μm diameter SPADs in 150 nm standard CMOS.

Some insight on the origin of DCR can be obtained by analyzing its temperature dependence. Figure 3 shows the DCR temperature dependence of four devices with the same area, representative of the behavior of a whole distribution. The three SPADs with high DCR have an activation energy lower than E_G_/2, indicating the presence of trap-assisted tunneling. For the SPAD with low DCR, the activation energy has a transition between E_G_ and 0.2 V as the temperature decreases. In this device, DCR is dominated by injection of minority carriers from the neutral regions at high temperatures, and from tunneling at low temperatures. The relative weight between the different components determine the shape of the DCR distribution. If the devices are small and the amount of contaminants is low, DCR will mostly be dominated by minority carrier injection or band-to-band tunneling, and will be very uniform. If, on the contrary, the amount of contaminants is relatively high, the distribution will not show a plateau.

The integration of SPADs in deep submicron technology nodes has several obvious advantages. On one hand, being an intrinsically digital device, the possibility of introducing in-pixel dense digital readout electronics such as counters or TDCs is appealing. On the other hand, it is increasingly clear that a doping profile customization is absolutely needed to obtain detectors with acceptable performance in advanced nodes. The first attempts to fabricate SPADs in unmodified 90 and 65 nm processes have led to devices with very high DCR, mainly due to tunneling [15]. In fact, the well doping concentration steadily increases with device scaling. As a general consideration, SPADs integrated in standard processes with no process modifications have hardly an optimized DCR.

A customization of the doping profiles is required to optimize the SPAD characteristics. On the one hand, DCR can be reduced by carefully tuning the electric field profile in the avalanche region [20], so as to minimize the contribution of tunneling. This is also an advantage if the device needs to be cooled, as the absence of tunneling provides a more efficient DCR reduction with decreasing temperature. In fact, once the doping profiles are optimized for tunneling reduction, the device DCR is mainly due to the contamination by heavy metals. Using both excellent starting material and a clean production line, as in most CIS processes, is fundamental for the production of low-noise devices. The use of advanced imaging processes, in fact, can lead to devices having good characteristics both regarding PDE and DCR, as was demonstrated in a few cases [14,21].

The electric field profile affects the PDE through the optimization of avalanche triggering probability. In a graded profile the breakdown probability reaches very large values at smaller voltages than in step profile junction with similar breakdown voltages [18]. A good PDE at low excess bias can thus be obtained by combining a graded profile with an optimized optical stack, which is readily available in a CIS process [14].

Afterpulsing is a source of correlated noise, and in time resolved applications it can lead to measurement distortions if it is not minimized. Since its origin lies in the presence of deep trapping centers, the quality of the process can also contribute to its reduction. In addition, a careful design of the quenching circuit, ensuring a minimum stray capacitance and fast avalanche extinction, helps keeping afterpulsing under control. In general, in small CMOS devices, the total afterpulsing rate can be maintained at acceptable levels with a few tens ns hold-off time [11,14,22]. One notable exception to this general observation is a 0.35 HV processes, which has a long tails and needs a hold-off time of 100s ns [23]. This peculiar behavior, limited to a single 0.35 HV process has been reported by several authors, but the reason of the long afterpulsing tail has not yet been completely understood.

Optical cross-talk is a critical parameter in SPAD arrays, arising from the emission of optical photons during an avalanche event [24]. Measurements on dense p+/nwell SPAD arrays have shown that cross-talk rates can be as high as a few percent for nearest neighbor devices if the fill factor is in the order of 70% [25]. For optimal performance, therefore, cross-talk should be minimized by introducing deep trench isolation, as currently done in silicon photomultiplier by most manufacturers [26].

### 2.3. Uniformity

**Figure 4:**
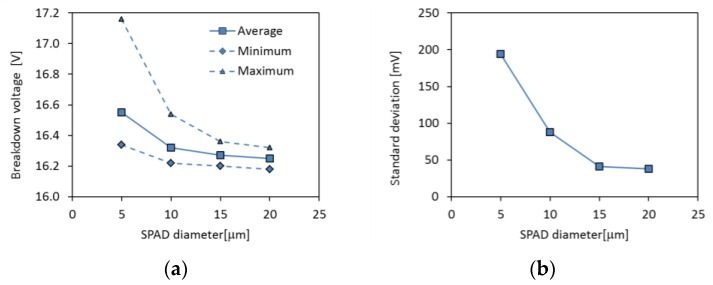
Measured breakdown voltage as a function of SPAD diameter. Measurements were done on 150-nm CMOS SPADs [27]. (**a**) Breakdown voltage distribution; (**b**) breakdown voltage non-uniformity (standard deviation).

The uniformity of breakdown voltage is of paramount importance for the integration of SPAD image sensors, since all the SPADs are biased at the same voltage. Non-uniformities in the order of 100 mV can be tolerated if SPADs are operated at excess bias voltages of a few volts. A good uniformity of PDE along the pixel array is also required in image sensors. Experimental investigations on 150 nm CMOS SPADs have shown that breakdown voltage non-uniformity is larger in small devices, reaching values in the order of 1 V for 5-μm diameter SPADs, while 10-μm devices have a peak-to-peak non-uniformity lower than 0.5 V and in larger ones it is in the order of 100 mV (Figure 4). The effect of device size is also visible in the average breakdown voltage. The trend is toward a decreasing average breakdown voltage with device area. Surprisingly, however, once the avalanche has been triggered, the PDE results very uniform in all the tested detectors, with non-uniformities lower than 1% [27].

### 2.4. Layout

**Figure 5:**
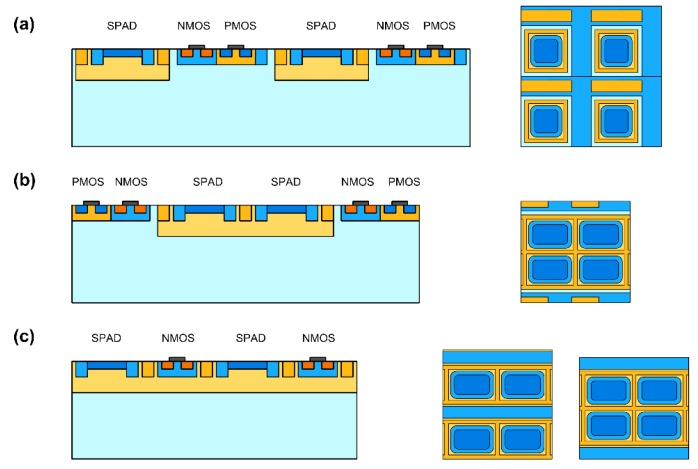
Cross section and 4 × 4 pixel layout of (**a**) SPADs integrated in separate deep nwell; (**b**) SPADs sharing the same deep nwell; (**c**) SPADs and NMOS transistors sharing the same deep nwell.

High fill factor pixel arrays can be obtained only with a careful minimization of guard ring and of the area occupied by in-pixel readout electronics. Deep-nwell sharing between different SPADs and between SPADs and electronic readout circuits can be used to effectively increase the device packing and therefore to help shrinking pixel size [28]. If every SPAD has a separate deep-nwell, as in Figure 5a, the bias can be applied either at the anode or at the cathode, and quenching circuitry can be connected to the other terminal. In the second case, the total device capacitance will be lower, but the distance between different devices should be maintained large enough to avoid punch through between the deep nwells. This layout solution was used in the early proof-of-concept arrays with large pitch and small fill factor [5,29].

Deep-nwell sharing can be exploited to reduce the dead area at the borders of the deep nwell, as shown in Figure 5b. With deep-nwell sharing, first proposed in [11] the cathode of all SPADs are in common and the quenching should be performed at the anode side. Densely-packed SPAD arrays can be easily obtained using currently available technologies if digital readout circuits are placed outside the deep nwell. High fill factors, limited only by the device guard ring, have been demonstrated, although the detectors were confined in arrays including only a few lines of SPADs [30,31,32,33].

The packing desity can be further increased if the readout n-type MOSFETs are included in the same deep nwell with the SPADs, as in Figure 5c. In this case, the pwell with the MOSFETs must be biased at a voltage close to the one applied to SPAD anode, and thus the breakdown voltage of the pwell/deep-nwell junction should be high enough to avoid early brekdown problems. The main disadvantage of this solution, which has been adopted in [19,34,35,36,37], is that p-type transistors cannot be used inside the pixel electronics.

## 3. Compact Pixels for Time-Resolved Imaging

The realization of photon-counting or time-stamping function within a given time window is straightforward with digital logic when using SPADs, since they provide a digital output. Although the fully digital solution meets the requirements of robustness and ease of implementation, the area occupation of the circuitry becomes extremely large. This is in contrast with the application field of this class of imagers, which is typically fluorescence lifetime imaging microscopy (FLIM), where also good spatial resolution and high efficiency are needed, driving towards small pixels with high fill factor.

### 3.1. Pixel Architectures

In order to keep the area small, analog solutions have been employed [19,35,36,37,38] by making use of analog counters and time-to-analog converters. The main techniques enabling a small pixel pitch are the implementation of the pixel circuitry using NMOS only so as to avoid nwells, the reuse of transistors for different purposes, a simple active-pixel readout with source follower and selection switch, and almost minimum-sized NMOS and capacitors, often making use of parasitic capacitances as storage nodes.

**Figure 6:**
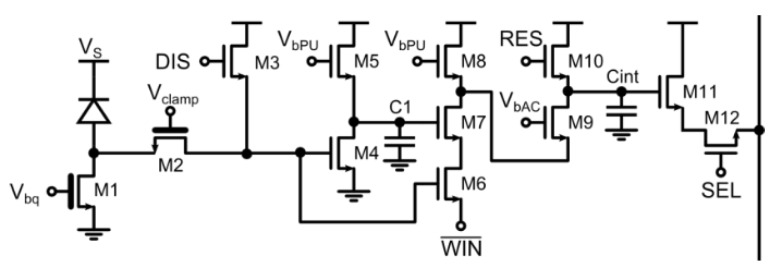
Time-gated pixel with analog counter, enabling gating windows from ≈1 ns up to several hundreds of milliseconds [19].

In [19] a pixel pitch of 25 μm with a 20.8% fill-factor was demonstrated in a 0.35 μm CMOS technology thanks to extensive use of analog techniques. The pixel schematic is depicted in Figure 6: the front-end is composed by a quenching transistor M1 and a clamp M2 which limits the voltage swing, and a disabling transistor M3. The gating circuit implements a pulse shortener by performing a logic AND between the SPAD pulse and its delayed and inverted companion, transmitting the WINn pulse to the analog counter. The latter signal defines the gating window, so when disabled ($\bar{WIN}$ = 1) no pulse is generated. The analog counter then discharges the integration capacitance by an amount proportional to the pulse width. This architecture reaches a gating window width down to 1 ns, but can also accommodate larger integration time, operating as a global shutter photon-counting pixel. Moreover, it can operate multiple gating cycles for a relatively long time, limited only by the charge leakage of the analog memory (hundreds of milliseconds): indeed, thanks to the decoupling effect of M7, the repeated activation of the $\bar{WIN}$ signal does not cause an output swing reduction due to charge injection.

The main issue of this pixel structure is the non-uniformity of the counting step in the array, which is driven by many mismatch constraints: the resulting pulse width of the gating circuit, mismatch of integration capacitances, distribution of the gating window signal, mismatch of the current limiter M9. In order to limit it to the measured value of 11.9%, non-minimum sized transistors have been used in the critical signal path although an unprecedented fill-factor was obtained.

Concerning the minimum time-gating, the precision for very small windows is limited by the pulse shortener: indeed, when a SPAD event occurs during the opening or closing of the gating window, the pulse may be sliced by the WINn signal and therefore a reduced step is recorded in the integration capacitance.

**Figure 7:**
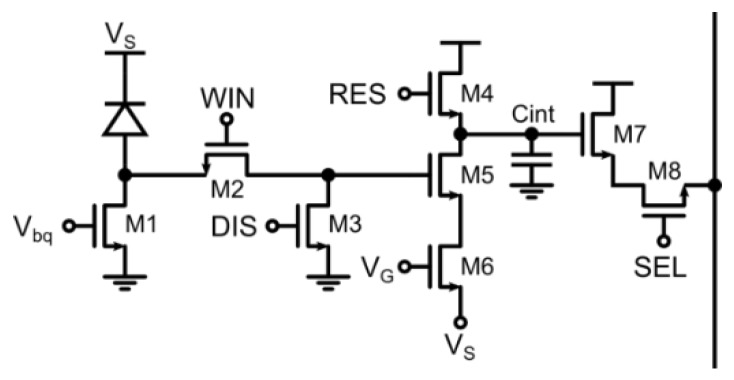
Analog counting pixel with coarse time gating for windows width larger than the SPAD dead time (*i.e.*, >10 ns) [35].

A strong reduction of the number of transistors per pixel has been achieved in [35] by replacing the gating circuitry with a single NMOS performing both clamping and time-gating. As shown in Figure 7, the SPAD is passively quenched and the pulse transmission can be inhibited by M2. The analog counter performs a controlled discharge of the integration capacitor through M5 and M6. The time gating performed by this circuit can only be coarse, meaning that it has to be larger than the dead time of the SPAD (typ. 10–100 ns) in order to have predictable voltage steps at the analog counter. Indeed, differently from the previous architecture, there is no pulse shortener and the full SPAD pulse has to be transmitted to the analog counter. Some small injection contribution is expected to impact on the output signal swing, due to WIN coupling through the gate-drain capacitance of M5 to the integration capacitor. Non-uniformities in this scheme are determined by mismatch between integration capacitors, current limiting transistor M6 but also differences in SPAD breakdown voltage and dead-time which change the shape of the pulse. Remarkable fill-factor of 26.8% in a 8-μm pitch is achieved in a 130 nm CIS technology. An on-chip 1-bit digital conversion has been implemented so as to achieve a fast single photon oversampled imager.

**Figure 8:**
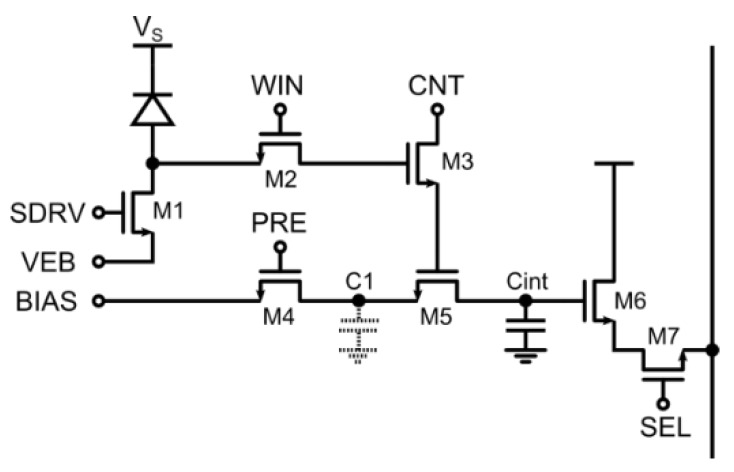
Analog time-gated pixel with transistor reuse and deferred analog counting [36].

Extensive reuse of transistors and a deferred counting technique has been implemented in [36] in order to further reduce the transistor count and at the same time allow for precise and short gating windows. This has been achieved at the expense of generality: in this implementation, no continuous integration is possible, but only periodic excitation/gating schemes are allowed. As shown in Figure 8 the frontend is composed by a switch M1 acting both as precharge and disable with independent gate and source voltages, while M2 clamps and samples the SPAD pulse. The main difference with respect to the previous schemes is that, due to the repetitive nature of the measurement, the counting operation is performed after the sampling of the SPAD state in the observation window. Again, counting is performed by subtracting a controlled amount of charge from an integration capacitor.

The deferred counting scheme makes the counting step independent from the SPAD pulse nature, width, and position within the gating time window, achieving sharp window edges down to 200 ps rise/fall time and 750 ns minimum gating window width. This advantage was exploited using almost minimum-sized transistors in order to obtain a pixel pitch of 15 μm and 21% fill-factor. At the same time, a self-referenced column-wise analog to digital conversion cancels the residual non-uniformity of 15.7% by using the very same pixel as a ramp generator for a single-slope ADC.

As a drawback, this scheme does not allow for more than one pulse to be counted for a single excitation/gating cycle, which is actually not a limitation when the gating window has to be smaller than the dead-time of a SPAD. For example, typical FLIM decay times fall within the range 1–10 ns, typically smaller than the SPAD recharge time. Another limitation is given by the fact that even if no events are recorded, the deferred counter is stimulated with the digital signals, and therefore charge is injected and accumulated, reducing the available output voltage swing thus limiting the dynamic range to few hundreds of counted photons between each readout.

Another function suitable for compact SPAD imaging arrays is the photon timestamping by using time-to-analog converters (TAC). In [38] a fairly complex TAC with in pixel digital conversion of the analog value was presented, but finally achieving a low fill-factor of 1% in a 50-μm pitch. Optimization of the area occupation can surely be improved by moving the conversion off-pixel, as described in [37]. With an approach similar to the analog photon counting described in the previous paragraphs, TAC circuitry can also be implemented using only NMOS transistors.

**Figure 9:**
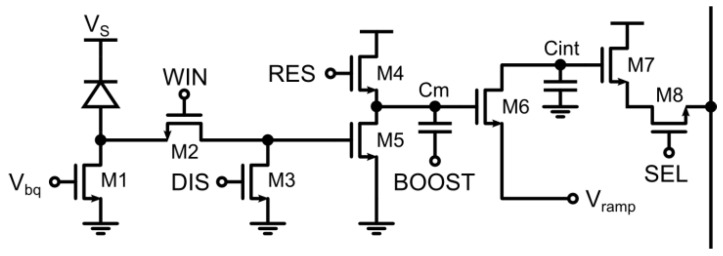
Compact NMOS-only TAC-based pixel [38].

In Figure 9 the schematic shows a frontend with a standard passive quenching with disabling circuitry, driving a dynamic memory composed by M4 and M5. Whenever a SPAD pulse occurs, the memory is fully discharged, sampling onto the storage capacitor the present reference voltage fed to the whole array, representing time in the analog domain. This solution virtually eliminates many of the non-uniformity sources affecting analog counters: among remaining issues impacting uniformity and linearity, there are leakage from the storage capacitor and proper distribution of the fast-varying reference signal to the whole array. A remarkable 20% fill-factor in a 8 μm pitch pixel is achieved, with an overall jitter of 368 ps rms.

### 3.2. Analysis and Comparison

The time-gated analog counters presented in [19] and [36] can be directly compared as they perform a very similar operation, with the main difference that the deferred counting scheme of [36] does not allow long integration time with global shutter.

**Figure 10:**
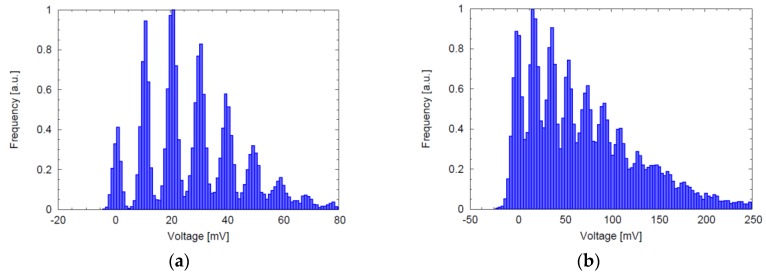
Pixel analog output histograms for the analog counting pixels of [19] and [36], depicted in (**a**) and (**b**), respectively.

As a first comparison, the analog photon counting may be addressed: by selecting a single pixel and recording the output analog values, it is possible to reconstruct an histogram showing the characteristic peaks corresponding to the detection of 0, 1, 2, …, photons. As shown in Figure 10, both histograms show clearly the single photon detection resolution, but Figure 10a highlights a better noise performance with almost isolated peaks. The higher noise visible in Figure 10b is due to the smaller in-pixel capacitance values used for the analog counting, leading to larger kTC noise contribution. In both cases, the kTC noise is repeatedly accumulated during the charge transfer, and progressively confuses individual photon peaks; anyway, this occurs when the shot noise is already the dominant noise source [36].

**Figure 11:**
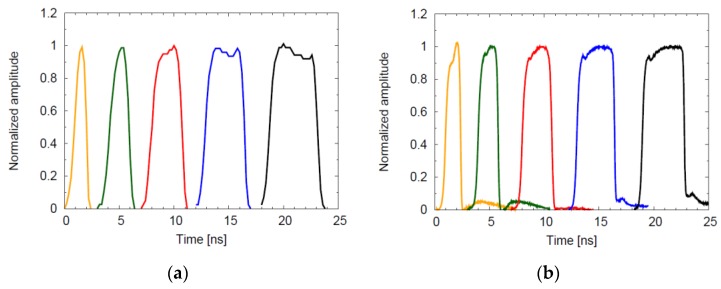
Time-gating windows for different widths for the analog counting pixels of [19] and [36], depicted in (**a**) and (**b**), respectively.

A second comparison can be made by exciting the sensor with a short laser pulse, in this case a 70-ps FWHM laser, progressively increasing the relative delay. The analog output then follows the shape of the gating window performed by the pixel. In Figure 11 this measurement has been repeated for windows width of approximately 1, 2, 3, 4, 5 ns; in both cases, the different window widths have been programmed to the internal logic which is stabilized against variations using a locked control loop with a reference clock. In this case it can be observed that edges of gating windows are better defined in Figure 11b: the motivation lays in the technique used to start and close the windows, avoiding the use of a monostable.

Table 1 summarizes the main parameters of all the analyzed SPAD image sensors, in particular for what concerns their compactness, size, and timing performance.

## 4. Discussion and Conclusions

The way towards high-resolution single-photon image sensors with timing capabilities (either implementing time-gating or time-stamping pixels) has to be pursued through two parallel approaches: on one side the optimization of the device, and on the other side with area-efficient circuit topologies.

The device optimization will inevitably go through the use of specialized process options, as already happened to conventional CIS processes. Indeed, SPAD devices will surely benefit from CIS (clean) processes with custom profile, which will enable reduction of tunneling and optimization of photon detection probability [39]. Additional modules, such as deep trench isolation in order to reduce cross talk, especially for deep-junction SPADs, will also improve the performance in densely packed arrays.

As far as pixel circuits is concerned, they must employ area-efficient analog and all-NMOS topologies in order to exploit shared nwell SPAD layout, passive quenching, transistors reuse, and possibly self-referenced conversion for increased reliability and readout speed in perspective of high-resolution imagers.

Finally, in the near future SPAD-based imagers could definetely take advantage of the advent of 3D-stacked fabrication technologies exploiting the co-integration of specialized CIS back-side illuminated sensing layer with deep-submicron digital CMOS. BSI-compatible SPADs offer higher sensitivity in the NIR region that combined with the stacking on advanced digital CMOS technologies will enable the realisation of pixel pitches in the order of a few micrometers and high fill factor [40,41]. At the same time efficient processing of the generated data flow will still be possible. These features, not only will drastically improve the performance of SPAD-based range cameras, but will open the way to new applications that are now dominated by other detector technologies, such as high dynamic range, high sensitivity and high speed intensity cameras.

## Figures and Tables

**Table 1:** Comparison between recent compact analog-based SPAD sensors for time-resolved imaging.

	[19]	[35]	[36]	[37]
Process	0.35 μm HV	0.13 μm CIS	0.35 μm HV	0.13 μm CIS
Supply	3.3 V	1.2 V	3.3 V	1.2 V
Array size	32 × 32	320 × 240	160 × 120	256 × 256
Pixel pitch	25 μm	8 μm	15 μm	8 μm
Fill-factor	20.8%	26.8%	21%	20%
NMOS per pixel	12T + 2C	8T + 1C	7T+1C	8T + 2C
Timing	Gating >1.1 ns	Gating > 10 ns	Gating > 0.75 ns	TAC > 0.37 ns
Interface	Analog	Analog/Digital 1 b	Analog/Digital 8 b	Analog/Digital 2 b
Consumption	33 mW	69.5 mW	20.6 mW ^a^	n.a.
			157 mW ^b^	

^a^ Analog readout mode; ^b^ Digital readout mode.
